# The Implementation and Appraisal of a Novel Confirmatory HIV-1 Testing Algorithm in the Microbicides Development Programme 301 Trial (MDP301)

**DOI:** 10.1371/journal.pone.0042322

**Published:** 2012-09-11

**Authors:** Ute Jentsch, Precious Lunga, Charles Lacey, Jonathan Weber, Janet Cairns, Gisela Pinheiro, Sarah Joseph, Wendy Stevens, Sheena McCormack

**Affiliations:** 1 School of Pathology University of the Witwatersrand, Johannesburg, South Africa; 2 MRC Clinical Trials Unit, London, United Kingdom; 3 University of York, York, United Kingdom; 4 Imperial College School of Medicine, London, United Kingdom; University of Nebraska Medical Center, United States of America

## Abstract

We describe the application of a novel HIV confirmatory testing algorithm to determine the primary efficacy endpoint in a large Phase III microbicide trial. 9385 women were enrolled between 2005 and 2009. Of these women, 537 (6%) had at least one positive HIV rapid test after enrolment. This triggered the use of the algorithm which made use of archived serum and Buffy Coat samples. The overall sample set was >95% complete. 419 (78%) of the rapid test positive samples were confirmed as primary endpoints using a combination of assays for the detection of HIV-specific antibodies (EIA's and Western Blot), and for components of the virus itself (PCR for the detection of nucleic acids and EIA for p24 antigen). 63 (12%) cases were confirmed as being HIV-positive at screening or enrolment and 55 (10%) were confirmed as HIV negative. The testing algorithm confirmed the endpoint at the same visit as that of the first positive rapid test in 90% of cases and at the time of the preceding visit in 10% of cases. Of the 63 cases which were subsequently confirmed to be HIV-1 positive at or before enrolment, 54 specimens contained no detectable HIV antibodies at screening or enrolment. However, 43 were positive using an EIA which detects both HIV antigen and antibody and also had a positive p24 antigen or HIV PCR test, which was highly suggestive of acute infection. There were 6 unusual cases which had undetectable HIV-1 DNA or RNA. In 4 of the 6 cases the presence of HIV-1-specific antibodies was confirmed by Western Blot. One of these cases with an indeterminate Western Blot was a previous vaccine trial participant. The algorithm served the objectives of the study well and can be recommended for use in determining HIV as an endpoint in clinical trials.

**Trial Registration:**

ISRCTN.org ISRCTN 64716212

## Introduction

Accurate and reliable detection of HIV infection is of obvious intrinsic benefit but is also crucial to ensure the validity and quality of clinical trials in which HIV infection is an endpoint. The Centre for Disease Control (CDC) criteria define HIV infection status on the basis of repeated reactive Enzyme Immunoassays (EIA) and a positive confirmatory Western Blot (WB) or Immunofluoresence Assay (IFA) for the detection of specific HIV antibodies [1]. This type of algorithm is commonly employed in countries where the prevalence of HIV is low (<1%).

CDC and World Health Organization (WHO) guidelines advocate the use of rapid tests in a clinic setting for the purposes of cost-effective patient management [1]–[5]. It is also recommended that newly diagnosed cases have a second sample collected and tested in order to exclude clerical or technical errors. P24 antigen (P24 Ag) testing and HIV viral load (VL) testing do not constitute primary diagnostic tests, but are useful supplementary tests in helping to resolve inconclusive serological results.

For settings in which resources are limited, the WHO advocates the use of serial rapid tests for the detection of HIV infection [3]. This applies to many parts of Africa, where the necessary infrastructure and skills required for laboratory based assays such as Western blotting and PCR are limited, and it is only feasible to use less technically demanding assays. Areas with the highest prevalence of HIV are frequently poor in resources and, provided assay performance has been verified, reactive results obtained using 2 different rapid tests is routinely used to indicate evidence of HIV infection.

“Parallel testing”, in which 2 rapid tests are carried out concurrently, has also been advocated [3]. This strategy for HIV screening is popular in the clinical trial setting as the method contains intrinsic quality control and the extent of result concordance can be used to evaluate accuracy. This approach can be used to trigger further evaluation of discordant results, as these may indicate very early seroconversion.

Many laboratories in Africa offer HIV testing using EIA. These methods can supplement the Rapid tests carried out in clinical settings both for confirmatory testing purposes or to resolve the results of inconclusive rapid tests. Third generation HIV EIAs (3^rd^ gen HIV EIA) detect HIV-specific IgM antibodies which typically develop 3–4 weeks after infection [6], [7]. More recently developed fourth generation HIV EIAs (4^th^ gen HIV EIA), detecting both free p24 Ag and Ag complexed with specific antibodies (Ag/Ab), have enabled detection of infection up to 2 weeks earlier [8], [9]. Ly et al. showed that this relatively superior performance can be explained by an ability to detect very low concentrations of P24 Ag [9]. Ag/Ab assays are now recommended for use in first-line screening in many areas of the world, although only one has been approved by the United States Food and Drug Administration (FDA) to date [10], [11] . There is concern about the performance of some EIAs [12]–[15] and rapid tests [16]–[18] currently in use in certain parts of Africa. False positive results have been reported, and these have been shown to be particularly associated with early generation assays and cross reactions with other infectious agents and immune responses to them.

In a recent study in adolescents in Tanzania, Everett and colleagues explored the basis for false positive results which they obtained using the 4^th^ generation Murex Ag/Ab EIA [15]. After accounting for clinical, sociological and immunological variables, conditional logistic regression showed that false positivity was strongly associated with levels of specific IgG antibodies against *Schistosoma spp* and also with Rheumatoid factor (RF) suggesting cross reactivity. Trypanosomiasis and Leishmaniasis have also been similarly associated with false positive serology [19], [20]. It is therefore important to evaluate assays locally, to use assays with acceptable sensitivity and specificity and to use more specific assays in a confirmatory algorithm.

It is now considered highly desirable to test for acute HIV infection; both for reasons of public health and individual patient management. The acutely infected population is believed to be a major driver of new infections, as the viral load is particularly high during early stages of infection before the viral set point is reached [21]. Currently, the inclusion of PCR assays for the detection of HIV RNA in plasma or HIV DNA in cells (Nucleic acid amplification -NAAT) is advised as there is a period of about 3 to 4 weeks after infection when HIV-1 specific antibodies are undetectable and HIV rapid or EIA tests are uninformative [22]–[25].

The detection of viral nucleic acid is more sensitive and cost-effective than the detection of p24 Ag [26], [27]. Although molecular assays are not considered as “gold standard” diagnostic assays for HIV diagnosis in adults by the CDC, they are playing an increasingly important role in the field of HIV diagnosis, especially for the detection of acute infection and/or the resolution of inconclusive antibody results.

MDP 301 was a phase 3, multi-centre, randomized, double-blind, placebo-controlled trial to evaluate the efficacy and safety of 0.5% and 2% PRO 2000/5 microbicide gels. The study commenced in October 2005 and was completed end of September 2009. This trial is registered at http://isrctn.org, number ISRCTN 64716212 (grant number G0100137).

The trial was conducted at 6 research institutions in Africa: 3 in South Africa (Johannesburg, Durban and Mtubatuba) and one each in Zambia (Mazabuka), Tanzania (Mwanza) and Uganda (Masaka). A cohort of 9385 HIV negative women were enrolled and followed up for 52 weeks at all sites, except in Uganda, where women were followed-up for 2 years. The primary objective of the trial was to assess the safety and efficacy of both 2% and 0.5% PRO2000 gel in reducing vaginally acquired HIV infection. The results of the study have been published [28]. In February 2008, the 2% arm was discontinued for reasons of no benefit, when data was analyzed by the independent data monitoring committee. In this paper the HIV endpoint data is presented and includes results from the 2% arm. An HIV endpoint was defined as having occurred during the trial when a participant was confirmed to be HIV uninfected at enrolment by having a negative HIV EIA and PCR test and subsequently determined as HIV positive according to the algorithm at any follow up visit on the basis of the testing of 2 different samples.

The Medical Research Council (MRC) Clinical Trials Unit in London contracted “Contract Laboratory Services” (CLS), linked to the School of Pathology of the University of the Witwatersrand in Johannesburg South Africa, to function as the central laboratory. The key functions of CLS were: HIV endpoint confirmation, overall quality assurance and Good Clinical and Laboratory Practise (GCLP) implementation at local laboratories.

## Methods

### Ethics Statement

The main paper for this study has already been published [28]: “The protocol was approved by local and national ethics committees, in all participating countries and in the UK. Authorisation was obtained from the national regulatory authority in all participating countries and the US Food and Drug Administration. Participants indicated their consent by signature or witnessed thumbprint.”

### Participants and design

MDP301 was a phase 3, randomised, double-blind, and parallel-group trial. Full details of trial design, sample size, research sites, study populations, study conduct including the randomization and masking and data underpinning the sample size calculations have been reported elsewhere [29]. Participants were enrolled by 6 research institutions in Africa (three in South Africa and one each in Tanzania, Uganda and Zambia). Details of eligibility are described elsewhere [28] but the importance of this analysis required participants to be HIV negative at enrolment.

For the purposes of this study, all data obtained from the algorithmic testing was captured in an Excel format. The statistical approach was based on working out percentages for the testing outcomes. The denominators were total number of enrolled participants per site. The data were held in a single centralized database, were analyzed descriptively and presented as proportions.

### HIV rapid testing and local sample storage

HIV screening was carried out using parallel rapid testing at all but two sites (Table 1).

**Table 1:** Summary of rapid tests used by MDP Centres.

Assay	Approval	Centre
Determine HIV 1/2 (Abbott Laboratories, Illinois, USA)	WHO	All sites
Uni-Gold HIV test (Trinity Biotech, Wicklow, Ireland)	WHO	JHB, Mtubatuba
OraQuick Advance HIV 1/2 (OraSure Technologies, Bethlehem USA)	FDA	Durban
Capillus HIV-1/HIV-2 (Trinity Biotech, Wicklow, Ireland)	WHO	Tanzania
Genie II HIV-1/HIV-2 (BioRad)	WHO	Zambia

Abbreviations: JHB – Johannesburg.

Tanzania was in the progress of validating rapid testing [30], and was still relying on laboratory based EIA testing for screening purposes when the study commenced. In Uganda confirmation of a single positive rapid test was done by HIV EIA and HIV WB testing due to the poor specificity of HIV rapid tests reported in that geographical location [31]. The criteria for the selection of suitable HIV rapid tests were that all sites had to use WHO [32] or FDA [33] approved HIV rapid tests that were validated in each site. Before the study commenced an HIV testing validation exercise was performed. All sites were subjected to testing of a 30 member blinded panel (15 positive and 15 negative samples) using their HIV rapid and EIA kits. This panel was prepared by the NICD (National Institute for Communicable Diseases, South Africa) and was characterized using a panel of HIV EIAs, rapid tests, HIV-1 WB for the detection of HIV 1/2 antibodies, and an EIA for p24 Ag testing. The study sites had to achieve a score of > = 29/30 to qualify for competence for on-site HIV rapid and EIA testing.

HIV rapid testing was conducted at screening, weeks 12, 24, 40 and 52 (and weeks 66, 76, 88, 100 and 104 in Uganda). Serum samples were obtained up to 6 weeks before enrolment, at enrolment and then at weeks 4,12,24,40 and 52 (and week 104 in Uganda) and stored at −20°C at the local laboratory repository. Buffy Coat (BC) was collected and stored at −70°C at enrolment, weeks 24, 40, 52 (for all sites except Uganda) and at week 104 (Uganda only), (Table 2).

**Table 2:** Schedule of visits and corresponding blood draws.

Week	−6	0	4	12	24	40	52	66^2^	76^2^	88^2^	100^2^	104^2^
Rapids	X			X	X	X	X	X	X	X	X	X
Serum storage	X	X	X	X	X	X	X^1^	X				X
BC storage		X			X	X	X	X				X

1 All sites except Uganda.

2 Uganda only weeks 66 through to 104.

**Figure 1:**
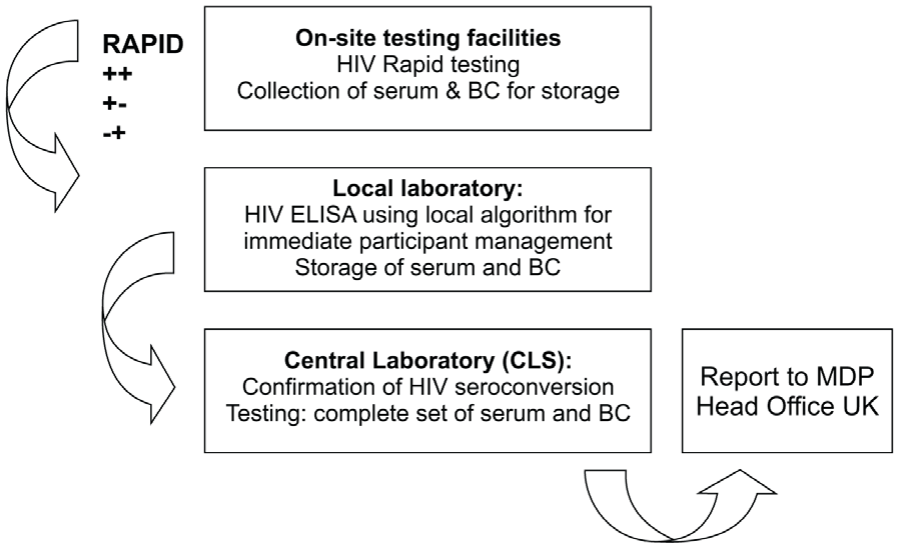
Overview of the HIV testing procedures. This figure describes the diagnostic tasks for the clinic, local laboratory and central laboratory.

Women who tested positive at screening had this status confirmed using the local country-specific algorithm and were classified as “not eligible” to join MDP301. Women with a positive or discordant rapid test after enrolment triggered the MDP 301 algorithm (Figure 1).

### Confirmation of HIV status

**Figure 2:**
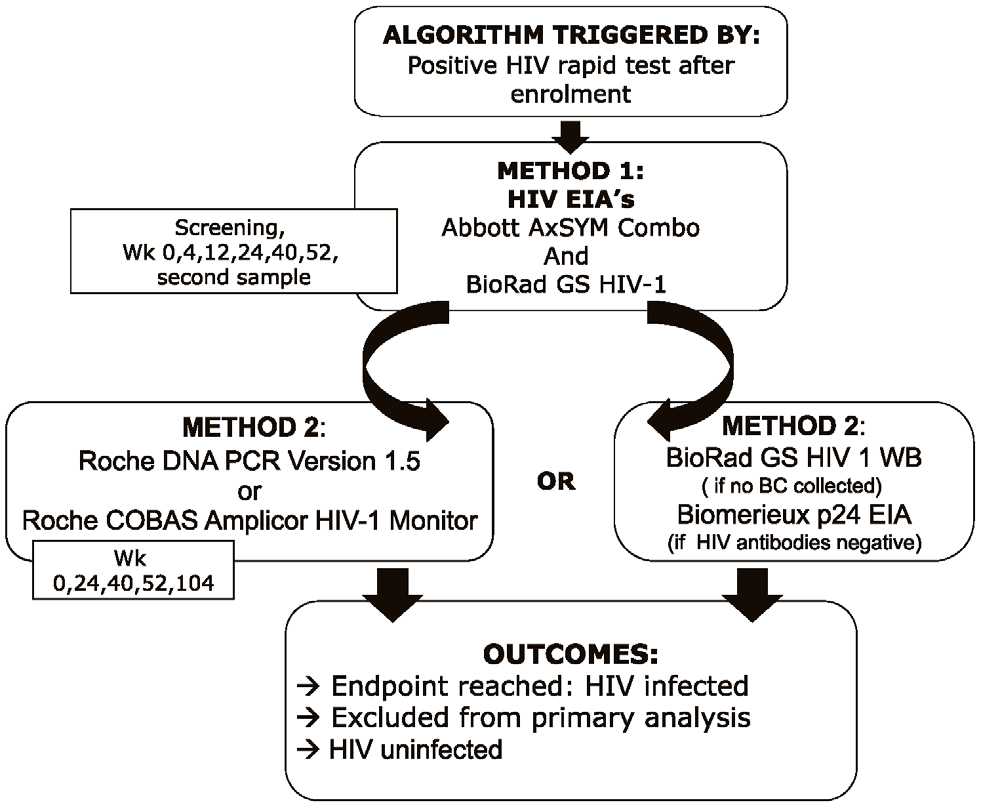
Confirmatory HIV testing algorithm used for MDP301. This figure details the diagnostic methods used at the central laboratory for all potential seroconverters and the possible outcomes.

The algorithm was triggered by a positive rapid test result at time after enrolment. The complete set samples (serum and BC) collected during any scheduled or unscheduled visit from screening up to and including the visit which triggered the algorithm were shipped on dry ice from the local laboratory repository to CLS (Figure 2). If HIV infection was confirmed using this set of samples, a second specimen was requested. This second (serum) sample was used to verify the seroconversion and was collected at the visit subsequent to the one which had triggered the algorithm.

MDP301 used a novel algorithm for the detection of HIV infection.The algorithm had to be able to confirm HIV infection based on two separate samples. At each endpoint, 2 different methods of diagnosis had to be available: 2 different EIAs and a confirmatory positive HIV qualitative DNA PCR, p24 EIA or HIV-1 WB. The WB was utilized as a second method of confirmation for those visits at which no BC was collected for DNA PCR (at week 4 and 12 for all sites; week 52, 76, for Uganda only). Where the BC was negative or failed to give a result (due to poor sample quality) a VL assessment was performed on the residual serum sample. The algorithm had to also allow for the detection of acute HIV infection to reliably diagnose those cases at or before enrolment.

At CLS, serum samples were tested for HIV-1 antibodies with Abbott AxSYM HIV Ag/Ab Combo (Wiesbaden, Germany), Bio-Rad HIV-1 Genetic Systems rLAV EIA (Redmond, USA), and Bio-Rad Genetic Systems HIV-1 Western Blot (Redmond, USA) assays. We used Biomerieux Vironostika HIV-1 antigen EIA (Boxtel, Netherlands) as a confirmatory assay for p24 testing. BC samples were tested for the presence of HIV-1 DNA using the Roche qualitative DNA PCR Version 1.5 assay (Roche Diagnostic Systems Inc, Branchburg, NJ, US). The Roche COBAS Amplicor HIV-1 Monitor (Roche Diagnostic Systems Inc, Branchburg, NJ, US) was used for the detection of HIV-1 RNA if the BC specimen was not satisfactory. A second serum sample was drawn for serological confirmation of the result at the next visit if HIV infection was indicated after the first round of testing.

The amplification of DNA over RNA was preferred because DNA is intrinsically more stable and easier to prepare and ship. In the South African setting this assay has a sensitivity and specificity of 99% and 99.8% respectively for HIV diagnosis in infants [34].

HIV seroconversion was defined as having occurred during the trial when a participant tested negative by rapid test, HIV EIA and PCR at enrolment and subsequently tested positive according to the algorithm at any follow up visit. An acute HIV infection was defined as a positive PCR or 4^th^ generation HIV EIA (Abbott AxSYM Combo) with a positive p24 result in the absence of HIV antibodies using the rapid test or 3^rd^ generation HIV EIA (Bio-Rad EIA). All results were peer reviewed by a panel of diagnostic experts, constituting the “HIV endpoint committee”. This included an independent expert, who was not involved in the study.

## Results

From October 2005 through to September 2009, samples from 537 participants were positive by rapid test at a follow up visit after enrolment. Over this period, 2946 serum samples and 1332 BC samples were shipped to CLS. No major problems were encountered with the shipping processes that could have impacted on sample viability. The samples were more than 95% complete: all 537 sets of samples were received in good condition. 17 of 419 expected second samples were not collected. This was typically because the participant was lost to follow-up. 6% (n = 74) of the BC samples were either inadequate (insufficient BC or a clotted sample) or because an incorrect sample type was collected. At one site for example, 40 plasma samples were collected instead of cells in error.

Of the 537 participants, who had positive rapid tests after enrolment, 66 were discordant and 471 were positive on both rapid tests. The stored sera and BC samples from all were tested according to the HIV confirmatory algorithm: 419 (78%) were confirmed as endpoints, 55 (10%) were confirmed to be HIV uninfected and 63 (12%) were confirmed as HIV infected at screening or enrolment (Table 3). A considerable number of participants at Africa Centre were found to be HIV infected prior to enrolment (n = 18, 24%) and this could not be explained by the existence of a longer enrolment window.

**Table 3:** Breakdown by site and outcome of the 537 samples which generated at least one positive rapid test result and were processed according to the MDP testing algorithm.

Site	Durban	JHB	Mtubatuba	Masaka	Mazabuka	Mwanza	Total
**Number (n)**	194	129	75	62	50	27	**537**
1Excluded	19 (10%)	13 (10%)	18 (24%)	10 (16%)	1 (2%)	2 (7%)	**63**
2Endpoint reached	144 (74%)	113 (88%)	46 (61%)	52 (84%)	47 (94%)	17 (63%)	**419**
3Endpoint not reached	31 (16%)	3 (2%)	11 (15%)	0 (0%)	2 (4%)	8 (30%)	**55**

1 HIV infected at screening or enrolment.

2 HIV infected after enrolment.

3 HIV uninfected.

### Uninfected cases

Of the 55 uninfected cases, 51 had a discordant rapid test result and in 3 cases both rapid tests were positive and in one case the rapid test was negative. The latter case was picked up as the sample was a random quality control sample that had an EIA test which gave a weakly positive result.

### False positive rapid results

55 of 537 cases which triggered the algorithm were not confirmed as endpoints. Mwanza generated the highest percentage of false positive rapid test results (n = 8, 30%) and this may have been due to errors in performing the assays as the site had just introduced the method. Numerically, most false positive rapids came from Durban (n = 31) and Africa centre (n = 11) sites, both sites located in KwaZulu Natal. The rate of false positive rapid tests in Durban and Africa Centre was significantly higher than the false positive rate from the Johannesburg site: 16% and 15% compared to 2%, respectively.

### Endpoints

Of the 419 confirmed endpoints, the algorithm detected seroconversion at the same visit as the rapid test in 83% (n = 349) of cases. For 16% (n = 68) of endpoints, the algorithm detected infection at an earlier visit. However, 28 of the 68 endpoints were acute infections and would not be expected to be detected by rapid tests which depend on the detection of HIV-specific antibodies. When these were excluded, the algorithm and rapid testing detected the endpoint at the same visit in 89% (349/391) of cases. In 2 cases a discordant rapid result was confirmed as positive at the following visit.

### Acute infections

There were 54 cases of acute HIV-1 infection. This was defined on the basis of being negative for the presence of HIV-1 specific antibodies but positive for the presence of HIV DNA or HIV RNA or p24 antigen. All these cases seroconverted at a later visit when antibodies could be detected using the conventional methods. 28 of the 63 (44%) seroconversions occurring before or at enrolment were acute cases.

43 of the 54 acute cases (80%) generated a positive HIV Combo EIA result that was confirmed by HIV PCR or p24 Ag testing. 11 had a negative HIV Combo result but had a positive PCR or p24 Ag test.

### Unusual cases

There were 6 cases that generated unusual and/or unexpected combinations of test results (Table 4).

**Table 4:** Unusual cases which generated “unexpected” sets of results using the algorithm.

Case	Positive EIA	WB	DNA PCR (week)	RNA PCR (week)	Endpoint
1	Week 24	Pos	Neg ( 0, 24)	<50 c/ml (2^nd^ )	Yes
2	Week 52	Pos	Neg (0,24,40, 2^nd^ )	<400 c/ml (52)	Yes
3	Week 52	Pos	Neg (0,24,40)	<400 c/ml (52)	Yes
4	Week 12, 52, 2^nd^	Ind	Neg (0, 2^nd^ )	<50 c/ml (2^nd^)	No
5	Week 24,40,52, 2^nd^	Ind	Neg (0,24,40,52)	Not done	No*
6	Week 24	Not done	Pos (0, 24)	<400 c/ml (0)	Yes

Abbreviations: Neg: negative; Pos: positive; Ind: indeterminate; c/ml: copies/ml; 2^nd^: second sample collected after positive EIA.

* Participant co-enrolled in an HIV vaccine trial.

Cases 1 to 3 demonstrated clear evidence of seroconversion and this was confirmed by WB for the detection of HIV- specific antibodies. However, PCR testing did not confirm infection. None the less, these 3 cases were defined as endpoints based on confirmation by WB testing.

Cases 4 and 5 generated positive EIA's with indeterminate non-evolving WB banding patterns and negative PCR. Case 5 was discovered to be co-enrolled into an HIV vaccine trial and this probably explained the existence of EIA/WB reactivity. This might also have explained the inconclusive results seen for Case 4 but could not be confirmed. Neither of these cases was classified as endpoints.

Case 6 was a week 24 seroconversion with a positive PCR result at enrolment. The HIV endpoint committee consensus was that the positive enrolment PCR result was due to a sample error; however a delayed seroconversion could not be excluded.

## Discussion

Accurate endpoint determination for HIV infection is important in the context of clinical trials. This analysis of the HIV seroconversions which occurred during MDP301 confirms that the testing algorithm was user-friendly, accurate and had the additional benefit of being able to retrospectively detect acute HIV infection. The HPTN035 trial also evaluated the efficacy of PRO2000 and a similar algorithm was employed [35]. HPTN differed in the following ways: confirmation was performed by WB on 2 separate specimens, while the MDP algorithm confirmed HIV infection with two methods: HIV EIA and HIV PCR testing or by Western Blotting. Both studies collected a second sample for confirmation. The CAPRISA algorithm [36] was different again and HIV infection was based on 2 separate positive PCR results. In all 3 studies, the algorithms were able to detect acute infections at enrolment, which is crucial for the accurate diagnosis of HIV endpoints, the primary outcome of the trials.

**The data was >95% complete** and this reflected good accounting of samples at all centres. Monthly sample reconciliation was performed and this allowed for real-time tracking of sample movement to the repository. In addition, each site was assessed annually and random sample storage checks were performed. These quality control procedures, including the use of a reputable courier, ensured completeness of the sample set.

Although sites were provided with a standard operating procedure for the collection and processing of BC samples; further verification of the methodology, by rehearsing the procedure prior to the start of the study, might have prevented inappropriate samples being collected at one of the study sites.

**Our data show that the algorithm** was able to make a reliable diagnosis of HIV infection status in 531 of the 537 cases with a positive rapid test at a follow-up visit confirming its accuracy.

This was facilitated by the fact that a complete set of serum and BC samples was tested for each potential endpoint from screening through to seroconversion, using 2 different methods of diagnosis plus testing of a second serum specimen. In addition, all cases were reviewed by the “HIV endpoint committee”.

**HIV rapid** testing correlated with confirmatory endpoint testing in 90% of cases. 51 of the remaining 55 cases (10%) had a discordant rapid result which was confirmed as negative for HIV by the algorithm. Inaccurate reading of rapid test results or technical operator related problems could not be excluded. Mwanza had just introduced rapid testing and so had relatively little experience of the technique which may have led to “over reading” and the relatively high rate of false positive results at this site. It has been pointed out previously in the literature that false positive rapid and EIA results resulting in suboptimal specificity are real concerns in areas with endemic tropical diseases, such as East African countries [15], [19], [20].

As it is essential to **exclude HIV infection at enrolment** in order to correctly assess efficacy of any product, the use of the HIV DNA PCR together with use of tests for the detection of HIV antibodies testing using the archived samples collected at enrolment was critical. This was reinforced by the relatively high number of seroconverters identified at screening and enrolment (n = 63, 12%). Testing of such a high proportion of samples at the first follow up visit provides evidence that these women were already seroconverting as they entered the study. Just under half (44%) of these were confirmed as acute infections.

The 4^th^ generation HIV EIA performed well for the diagnosis of all acute infections, detecting 80% of them, confirming the finding of Ly et al [9]. Branson [10] and Skidmore et al [37] also recommend the use of this assay for improved HIV screening outcomes and earlier detection of HIV, especially of acute cases and in settings of delayed seroconversion. These assays are therefore an appropriate tool for HIV screening in clinical trials. It would have been too costly to screen all women by HIV PCR for early HIV infection at enrolment, but testing all women at enrolment with a 4^th^ generation EIA would have enabled early diagnosis and potential intervention as well as exclusion from the trial. It is likely that the future of HIV testing for clinical trials will include a point-of-care HIV Ag/Ab and RNA Rapid assay.

**Unusual diagnostic results of HIV infection** have been previously described, including delayed seroconversion and negative HIV PCR results [37]–[41].

During MDP301 we have described cases which were confirmed to have seroconverted but which were negative for viral RNA and DNA using the specified assays. It is possible that these individuals were infected by clades not detected by the Roche assay or that these discrepancies were due to technical errors. Jackson et al [42] have previously found the Western Blot to be more reliable for confirming HIV infection.

In the latter study 94% of patients with AIDS had HIV DNA positive pellets using the Roche HIV-1 AMPLICOR test. However, on repeat testing of the same pellet a positive test DNA test was obtained.

As demonstrated in Case 5, it is essential to ask participants if they have or currently are participating in an HIV vaccine trial. Vaccine-induced seropositivity (VISP) has been described by Cooper et al in the HVTN trials [44]. Approximately 42% of HIV uninfected participants who had previously participated in HIV vaccine trials were positive for HIV-specific antibodies. Apart for possible problematic diagnostic and social consequences, these antibodies may persist for many years [43]. The rate of VISP varied significantly with the assay used. Where an HIV WB was performed, 10% of those who had been vaccinated had a positive WB and 66% had an indeterminate WB.

Delayed seroconversion may have accounted for the results seen for case 6, although the endpoint committee felt that a sample error had occurred at enrolment.

We have shown that study samples can be reliably stored and retrieved at local laboratories repositories in African study sites before shipping to the central Laboratory. Lessons learnt included the essential need for close monitoring of the sample preparation and storage at the beginning of the study and ongoing sample storage checks.

In conclusion, this study demonstrated that HIV endpoint testing for MDP301 was linked to an accurate, robust, user friendly algorithm in which all results were independently reviewed. Similar algorithms can be recommended for microbicides studies where HIV infection is the endpoint.

## References

[1] CDC (2001) Revised Guidelines for HIV Testing, Counselling and Referral. MMWR 50 (RR19): 1–58. 11718472

[2] WHO (1997) Revised recommendations for the selection and use of HIV antibody tests. Wkly Epid Rec 12: 81–88. 9238418

[3] WHO (2004) Rapid HIV Tests: Guidelines for use in HIV testing and counselling services in resource-constrained settings. ISBN 92 4 159181 1.

[4] CDC (2006) Revised Recommendations for HIV Testing of Adults, Adolescents, and Pregnant Women in Health Care Settings. MMWR 55 (RR14) 1–26. 16988643

[5] Delaney KP, Branson BM, Uniyal A, Phillips S, Candal D, et al (2011) Evaluation of the Performance Characteristics of 6 Rapid HIV Antibody Tests. Clin Infect Dis 52: 257–263. 21288853 10.1093/cid/ciq068

[6] Busch MP, Satten GA (1997) Time course of Viraemia and Antibody Seroconversion following Human Immunodeficiency Virus Exposure. Am J Med 102 (5B) 117–124. 9845513 10.1016/s0002-9343(97)00077-6

[7] Hecht FM, Busch MP, Rawal B, Webb M, Rosenberg E, et al (2002) Use of laboratory tests and clinical symptoms for identification of primary HIV infection. AIDS 16: 1119–1129. 12004270 10.1097/00002030-200205240-00005

[8] Ly TD, Laperche S, Couroucé AM (2001) Early Detection of Human Immunodeficiency Virus Infection Using Third- and Fourth-Generation Screening Assays. Eur J Clin Microbiol Infec Dis 20: 104–110. 11305462 10.1007/s100960000430

[9] Ly TD, Laperche S, Brennan C, Vallari A, Ebel A, et al (2004) Evaluation of the sensitivity and specificity of six HIV combined p24 antigen and antibody assays. J Virol Methods 122: 185–194. 15542143 10.1016/j.jviromet.2004.08.018

[10] Branson BM (2010) The Future of HIV Testing. J Acquir Immune Defic Syndr 55: S102–105. 21406978 10.1097/QAI.0b013e3181fbca44

[11] Food and Drug Administration (2010) Approval Letter - ARCHITECT HIV Ag/Ab Combo. http://www.fda.gov/BiologicsBloodVaccines/BloodBloodProducts/ApprovedProducts/LicensedProductsBLAs/BloodDonorScreening/InfectiousDisease/ucm216300.htm.

[12] Bredberg-Rådén U, Kiango J, Mhalu F, Biberfeld G (1988) Evaluation of commercial immunoassays for anti-HIV-1 using East African sera. AIDS 2: 281–285. 3140834 10.1097/00002030-198808000-00007

[13] Behets F, Disasi A, Ryder RW, Bishagara K, Piot P, et al (1991) Comparison of Five Commercial Enzyme-linked Immunosorbent Assays and Western Immunoblotting for Human Immunodeficiency Virus Antibody Detection in Serum Samples from Central Africa. J Clin Micro 29: 2280–2284. 10.1128/jcm.29.10.2280-2284.1991 PMC270313 1939584

[14] Everett DB, Weiss HA, Changalucha, Anemona A, Chirwa T, et al (2007) Low specificity of the Murex fourth-genenration HIV enzyme immunoassay in Tanzanian adolescents. Trop Med and Intern Health 12: 1323–1326. 10.1111/j.1365-3156.2007.01933.x 17949396

[15] Everett DB, Baisley KJ, McNerney R, Hambleton I, Chirwa T, et al (2010) Association of Schistosomiasis with False Positive HIV Test Results in an African Adolescent Population. J Clin Micro 48: 1570–1577. 10.1128/JCM.02264-09 PMC2863920 20181896

[16] Claasen M, van Zyl GU, Korsman SNJ, Smit L, Cotton MF, et al (2006) Pitfalls in rapid HIV antibody testing in HIV-infected children in the Western Cape, South Africa. J Clin Virol 37: 68–71. 16875874 10.1016/j.jcv.2006.06.008

[17] Madhivanan P, Krupp K (2007) Technological challenges in diagnosis and management of HIV infection in resource limited settings. BMJ 335: 165–166. 17656506 10.1136/bmj.39275.457188.AE PMC1934473

[18] Bruzzone B, Bisio F, Ventura A, Nigro N, Miguel LM, et al (2008) HIV serologic screening in a population of pregnant women in the Republic of Congo: suitability of different assays. Trop Med Int Health 13: 900–903. 18482195 10.1111/j.1365-3156.2008.02090.x

[19] Lejon V, Ngoyi DM, Ilunga M, Beelaert G, Maes I, et al (2010) Low specificities of HIV Diagnostic Tests Caused by Trypanosomiasis brucei gambiense Sleeping Sickness. J Clin Micro 48: 2836–2839. 10.1128/JCM.00456-10 PMC2916589 20573878

[20] Salinas A, Gorgolas M, Fernandez-Guerrero M (2007) Refrain from telling bad news: patients with leishmaniasis can have false positive HIV test results. Clin Infect Dis 45: 139–140. 17554722 10.1086/518709

[21] McMichael AJ, Burrow P, Tomaras GD, Goonetilleke N, Haynes BF (2010) The immune response during acute HIV-1 infection: clues for vaccine development. Nature Reviews Immunology 10: 11–23. 10.1038/nri2674 PMC3119211 20010788

[22] Morandi P-A, Schockmel GA, Yerly S, Burgisser P, Erb P (1998) Detection of Human Immunodeficiency Virus Type 1 (HIV-1) RNA in Pools of Sera Negative for Antibodies to HIV-1 and HIV-2. J Clin Microbiol 36: 1534–1537. 9620372 10.1128/jcm.36.6.1534-1538.1998 PMC104872

[23] Quinn TC, Brookmeyer R, Kline R, Shepherd M, Paranjape R, et al (2000) Feasibility of pooling sera for HIV-1 viral RNA to diagnose acute primary HIV-1 infection and estimate HIV incidence. AIDS 14: 2751–2757. 11125894 10.1097/00002030-200012010-00015

[24] Pilcher CD, McPherson JT, Leone PA, Smurzynski M, Owen-O'Dowd J, et al (2002) Universal Screening for Acute HIV Infection in a Routine HIV Counselling and Testing Population. JAMA 288: 216–221. 12095386 10.1001/jama.288.2.216

[25] Pilcher CD, Eron JJ, Gavin S, Gay C, Cohen MS (2004) Acute HIV revisited: new opportunities for treatment and prevention. J Clinl Invest 113: 937–945. 10.1172/JCI21540 PMC379335 15057296

[26] Henrard DR, Phillips J, Windsor I, Fortenberry D, Korte L, et al (1994) Detection of human immunodeficiency virus type 1 p24 antigen and plasma RNA: relevance to indeterminate serologic tests. Transfusion 34: 376–380. 8191559 10.1046/j.1537-2995.1994.34594249046.x

[27] Schacker T, Collier AC, Hughes J, Shea T, Corey L (1996) Clinical and Epidemiologic Features of Primary HIV Infection. Ann Intern Med 125: 257–264. 8678387 10.7326/0003-4819-125-4-199608150-00001

[28] McCormack S, Ramjee G, Kamali A, Rees H, Crook AM, et al (2010) PRO2000 vaginal gel for prevention of HIV-1 infection (Microbicides Development Programme 301): a phase 3, randomised, double-blind, parallel-group trial. Lancet 2010, published online DOI:10.1016/S0140-6736(10)61086-0. 10.1016/S0140-6736(10)61086-0 PMC2956883 20851460

[29] Nunn A, McCormack S, Crook AM, Pool R, Rutterford C, et al (2009) Microbicides Development Programme; design of a Phase III trial to measure the efficacy of the vaginal microbicide PRO2000/5 for HIV prevention. Trials 10: 99. 19860888 10.1186/1745-6215-10-99 PMC2774685

[30] Everett DB, Kathy B, Changalucha J, Vallely A, Watson-Jones D, et al (2009) Suitability of Simple Human Immunodeficiency Virus Rapid Tests in Clinical Trials in Community-Based Clinic Settings. J Clin Microbiol 47: 1058–1062. 19244458 10.1128/JCM.01998-08 PMC2668306

[31] Gray RH, Makumbi F, Serwadda D, Lutalo T, Nalugoda F, et al (2007) Limitations of rapid HIV-1 tests during screening for trials in Uganda: diagnostic test accuracy study. BMJ| ONLINE FIRST bmj.com 335: 1–4. 10.1136/bmj.39210.582801.BE PMC1934458 17545184

[32] WHO, UNAIDS (2002) HIV Simple/Rapid Assays: Operational Characteristics (Phase 1) Report 12, Whole blood specimens.

[33] CDC (2010) FDA Approved Rapid HIV Antibody Screening Tests. http://www.cdc.gov/hiv/topics/testing/rapid/rt-comparison.htm

[34] Stevens W, Erasmus L, Moloi M, Taleng T, Sarang Somaya (2008) Performance of a Novel Human Immunodeficiency Virus (HIV) Type 1 Total Nucleic Acid-Based Real-Time PCR Assay Using Whole Blood and Dried Blood Spots for Diagnosis of HIV in Infants. J Clin Micro 46: 3941–3945. 10.1128/JCM.00754-08 PMC2593254 18923017

[35] Abdool Karim SS, Richardson BA, Ramjee G, Hoffman IF, Chirenje ZM, et al (2011) Safety and effectiveness of vaginal microbicides BufferGel and 0,5% PRO 2000 Gel for the prevention of HIV infection in women. AIDS 25: 957–966. 21330907 10.1097/QAD.0b013e32834541d9 PMC3083640

[36] Abdool Karim Q, Abdool Karim SS, Frohlich JA, Grobler AC, Baxter C, et al (2010) Effectiveness and Safety of Tenofovir Gel, an Antiretroviral Microbicide, for the Prevention of HIV Infection in Women. CAPRISA. Science Express 1–13. 10.1126/science.1193748 PMC3001187 20643915

[37] Skidmore S, Devendra S, Weaver J, Short J, Osman H, et al (2009) A case study of delayed HIV-1 seroconversion highlights the need for Combo assays. Int J STD AIDS 20: 205–206. 19255273 10.1258/ijsa.2008.008263

[38] Jenny-Avital ER, Beatrice ST (2001) Erroneously low or undetectable plasma human immunodeficiency virus type 1 (HIV-1) ribonucleic acid load, determined by polymerase chain reaction, in West African and American patients with non-B subtype HIV-1 infection. Clin Infect Dis 32: 1227–1230. 11283814 10.1086/319752

[39] Zaman MM, Recco RA, Haag R (2002) Infection with Non-B Subtype HIV Type 1 Complicates Management of Established Infection in Adult Patients and Diagnosis of Infection in Newborn Infants. Clin Infect Dis 34: 417–418. 11774090 10.1086/323186

[40] Trezi R, Niero F, Iemoli E, Capetti A, Coen M, et al (2007) Late HIV seroconversion after non-occupational postexposure prophylaxis against HIV with concomitant Hepatitis C virus seroconversion. AIDS 21: 262–263. 17197828 10.1097/QAD.0b013e328011922c

[41] Bøgh M, Machuca R, Gerstoft J, Pedersen C, Obel N, et al (2001) Subtype-specific problems with qualitative Amplicor HIV-1 DNA PCR test. J Clin Virol 20: 149–153. 11166664 10.1016/s1386-6532(00)00147-5

[42] Brooks Jackson J, Parsons JS, Nichols LS, Knoble N, Kennedy S, et al (1997) Detection of Human Immunodeficiency Virus Type 1 (HIV-1) Antibody by Western Blotting and HIV-1 DNA by PCR in Patients with AIDS. J Clin Microbiol 35: 1118–1121. 9114392 10.1128/jcm.35.5.1118-1121.1997 PMC232714

[43] Silbermann B, Tod M, Desaint C, Pialoux G, Petitprez K, et al (2008) Long-Term Persistence of Vaccine-Induced HIV Seropositivity among Healthy Volunteers. AIDS Res Hum Retroviruses 24: 1–4. 19000023 10.1089/aid.2008.0107

[44] Cooper CJ, Metch B, Dragavon J, Coombs RW, Baden LR (2010) Vaccine-Induced HIV Seropositivity/Reactivity in Noninfected HIV Vaccine Recipients. JAMA 304: 275–283. 20639561 10.1001/jama.2010.926 PMC3086635

